# Implementing a psychosocial care approach in pediatric inpatient care: process evaluation of the pilot Child Life Specialist program at the University Hospital of Munich, Germany

**DOI:** 10.3389/fped.2023.1178871

**Published:** 2023-06-07

**Authors:** Julia Hummel, Stephan Voss, Holly Clark, Michaela Coenen, Christoph Klein, Eva A. Rehfuess, Valerie Zu Rhein, Varinka Voigt-Blaurock, Caroline Jung-Sievers

**Affiliations:** ^1^ Department of Pediatric Surgery, Dr. von Hauner Children’s Hospital, University Hospital, Ludwig-Maximilians-University (LMU) Munich, Munich, Germany; ^2^ Chair of Public Health and Health Service Research, Institute of Medical Data Processing, Biometrics and Epidemiology (IBE), Faculty of Medicine, LMU Munich, Munich, Germany; ^3^ Pettenkofer School of Public Health, Munich, Germany

**Keywords:** child life specialist, complex intervention, influencing factors, logic model, pediatric psychosocial care, process evaluation, qualitative interviews

## Abstract

**Background:**

Child Life Specialists (CLSs) are psychosocial care professionals of child development and health who focus on the individual needs and rights of young patients. CLSs accompany sick children and focus on the children's perspective and their reality of life. CLS programs are already established in clinical settings in the United States and other Anglophone countries but have not yet been piloted in the German health care setting, neither has their implementation been evaluated in this context. This study aimed to explore the factors influencing the implementation of a pilot CLS program in pediatric inpatient care at the Dr. von Hauner Children's Hospital at the University Hospital of Munich, Germany.

**Methods:**

Building on methods commonly employed in the evaluation of complex interventions, we developed a logic model to guide the process evaluation of our program. Semi-structured interviews with four groups of stakeholders were conducted in person or via videoconferencing between June 2021 and January 2022. Data was analyzed collectively using the method of qualitative content analysis by Mayring.

**Results:**

Fifteen individual interviews were conducted with patients (children aged 5–17 years, *n* = 4), parents (*n* = 4), CLSs (*n* = 4) and other health professionals (*n* = 3). Factors influencing the implementation were identified on three levels: system, staff and intervention. On the system level, a clearer definition of CLSs’ tasks and responsibilities was perceived as important and would likely lead to a delineation from other (psychosocial) professions and a reduction of potential resistances. On the staff level, lacking training opportunities and feelings of being insufficiently skilled were limiting the CLSs professional self-confidence. On the intervention level, the emergence of a unique characteristic of the CLSs’ work (i.e., preparation for medical procedures) supported the acceptance of the new program.

**Conclusions:**

The implementation of a CLS program into an established hospital system with existing psychosocial care services is challenging. Our results contribute to a better understanding of implementation processes of such an additional psychosocial care approach and provide recommendations for addressing upcoming challenges.

## 1. Introduction

A hospital stay can be a challenging and stressful situation for children. Leaving their home and familiar surroundings, dealing with illness, and potentially experiencing pain- and anxiety-inducing procedures are only some of the numerous stressors children face in hospitals. Anxiety and stress symptoms during hospitalization can disrupt children's development and lead some to experience posttraumatic stress symptoms after they have been discharged (1–4). Children with mental health problems during hospitalization (e.g., depression, substance abuse) have a higher risk of being readmitted to hospital (5) which may further exacerbate the children's mental burden. Thus, the psychosocial care of hospitalized children which aims to respect their needs and which supports the children's ability to cope with hospital experiences is of utmost importance.

One approach to increasingly embrace the children's perspectives in pediatric health care is the implementation of Child Life Specialist (CLS) programs. CLSs are psychosocial care professionals of child development and health who strive to change perspectives in the hospital system by focusing on the needs and rights of children and respecting their individual reality of life. CLSs accompany and support children during their hospital stay and integrate children's perspectives into processes and structures in hospitals (6). CLSs seek to normalize the hospital environment by educating children about their diagnosis and preparing them for medical procedures. Possible CLSs’ interventions encompass providing important (medical) information in a child-friendly and age-appropriate manner, reenacting medical procedures, and teaching coping strategies, which the children can then utilize during stressful situations (7). CLSs collaborate in a highly interdisciplinary manner with physicians, nurses, psychologists, and other hospital employees in order to support the well-being of physically sick children in hospitals (8, 9). Thereby, CLSs particularly focus on the children's perspective, i.e., what children wish and need from their individual point of view. Structural constraints, such as lack of time and personnel, often lead health professionals to take on an adults’ perspective, i.e., during their work they usually view and evaluate their environment from the perspective of adults (10). In pediatric health care, a conscious shift from the adults’ perspective to the children's perspective is urgently needed.

In the United States and other Anglophone countries, pediatric psychosocial care is usually covered by psychologists, social workers, specialized nurses etc. as well as CLSs. Pediatric psychosocial care professionals are named differently in different countries, e.g., the term “Child Life Specialist” is used in the United States and Canada, whereas in the United Kingdom and New Zealand the term “Hospital Play Specialist” is more common (11). However, these professions are based on similar principles and can be united by the wide range of specific interventions that they deliver (7). In Germany, up to date no CLS programs exist and this approach is relatively unknown. Standard pediatric psychosocial care in German children's hospitals usually involves an interdisciplinary team of psychologists, educators, social workers and related professions, but is financially secured only in pediatric oncology and neonatology (12). Recent trends of economization in pediatric health care additionally reinforce critical gaps in the psychosocial care of children in hospitals, particularly in regard to considering children's needs and their individual perspective in all clinical processes and structures (10). To address this gap, starting in 2020 the first CLS program in Germany was piloted at the *Dr. von Hauner Children's Hospital,* the children's hospital and polyclinic at the *University Hospital of the Ludwig-Maximilians-Universität (LMU)* in Munich*.* The Dr. von Hauner Children's Hospital is one of the biggest children's hospitals in Germany with 108 beds and approximately 5,000 inpatients per year (13). It provides pediatric health care for patients from the metropolitan area of Munich and beyond (Bavaria, Germany and other countries).

To date, there is limited research on the effectiveness of CLS interventions. A systematic review showed that few controlled studies on the effects of CLS interventions exist (14). These studies report positive effects of CLS interventions on the reduction of fear (15), pain (16) and stress (17) in hospitalized children. However, heterogeneity regarding outcomes, procedures and quality is high (14). Specifically, included studies differed greatly in (i) the variables that were used as outcome measures, (ii) interventions that were provided by CLSs, and (iii) the risk of bias to be assumed. To the best of our knowledge, there are no studies examining the implementation of CLS programs and associated processes. In light of the limited evidence and to support implementation, longer-term institutionalization and scale-up of the CLS program in the German health care setting, a process evaluation of the pilot CLS program in Munich was conducted.

The aim of this study was to understand the factors influencing the implementation of a pilot CLS program in a German pediatric inpatient care setting based on qualitative interviews with different groups of stakeholders (patients, parents, CLSs, and other health professionals).

## 2. Material and methods

### 2.1. Preparatory phase: literature review and logic model development

The evaluation concept was based on the guidance of the Medical Research Council (MRC) on “developing and evaluating complex interventions” (18). The CLS program in Munich was regarded a complex intervention comprising multiple components in a complex organizational hospital setting: various services offered by CLSs, multiple interactions with patients, families, and hospital employees, and different outcomes targeted by CLSs (18, 19). Following the MRC framework, we would consider our evaluation project to be in the “feasibility phase” in which the intervention and the evaluation design are piloted in order to decide about the next stages of the project (18, 20). We first searched for and summarized existing literature on CLS programs and associated effects. We then reviewed what was known about the tasks of CLSs, the implementation of CLS programs as well as the assessment of CLSs’ effects. Up to 2021, the literature on CLS mainly consisted of few clinical reports and studies. Therefore, we conducted a systematic review on the effects of CLS interventions from randomized controlled trials (RCTs) on fear, pain and stress of children in hospitals (14).

**Figure 1:**
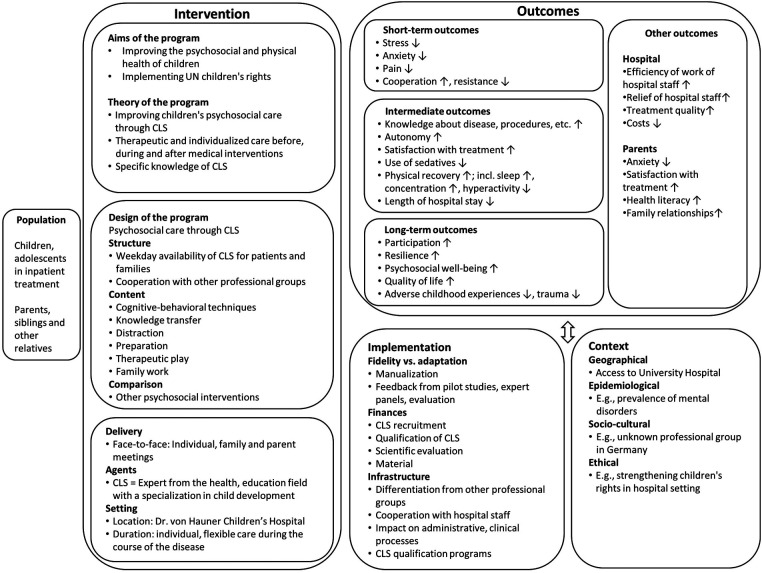
A-priori, system-based logic model of the CLS program.

Based on these findings, we developed an a-priori, system-based logic model (21) of the CLS program to establish a theoretical understanding of the program, its components and mechanisms at the beginning of the implementation and the research process (Figure 1). According to Anderson et al., “a logic model is a graphic description of a system and is designed to identify important elements and relationships within that system” (22). A-priori, system-based logic models can be used to visualize complex interventions according to the PICO framework (population, intervention, comparison, and outcome), and depict details of the implementation and the context an intervention is implemented in (21). Additionally, logic models (and the process of their development) can facilitate communication among stakeholders and researchers by providing a common understanding of an intervention (21). In our research, the logic model represents our understanding of the intervention at the beginning of its implementation and was used for methodological decisions, i.e., use of qualitative methods, selection of interview partners, development of interview guide etc. (18).

### 2.2. Focus of this study: process evaluation

A process evaluation examines mechanisms during the implementation of an intervention, takes a closer look if and how an intervention reaches its goals, or explores which factors contribute to or interfere with an intervention's success (18, 23). In line with the MRC guidance, we aimed to capture experiences with the CLS program and its implementation from the perspectives of different stakeholders (i.e., those being part of the CLS program or those being affected by it). Therefore, we chose a qualitative descriptive design for the process evaluation comprising semi-structured interviews and a qualitative content analysis following Mayring (24). According to the MRC guidance, “process evaluation” describes the strategy used for evaluating complex interventions, whereas qualitative methods describe the design of a process evaluation (23).

This study was conducted by researchers of the Chair of Public Health and Health Services Research at LMU Munich. The ethical standards of the Declaration of Helsinki (25) were taken into account when planning and conducting the study. Ethical approval was obtained by the Ethics Committee of the Medical Faculty of LMU Munich (no. 21-0435).

#### 2.2.1. Description of the CLS pilot

This study was conducted at *Dr. von Hauner Children's Hospital,* the children's hospital and polyclinic at the *University Hospital of LMU* in Munich*,* one of the biggest pediatric hospitals in Germany. The pilot CLS program was initiated in May 2020 by the hospital's director and the leader of the hospital's psychosocial team. The latter interned at an established CLS program in the United States before starting the implementation in Germany. Additionally, regular exchange meetings with leaders of CLS programs in the United States were installed. Four CLSs (full-time positions) with different professional backgrounds (children's nurses, psychologists, occupational therapists) were employed as psychosocial health care providers. Until January 2022 three more part-time positions were added (teacher, pedagogue, children's nurse). All of them were recruited internally within the hospital as well as externally. Since in Germany at that time no institutionalized training opportunities for the CLS profession existed, nor were there any German-speaking CLSs who could assist, CLSs were trained by a Certified Child Life Specialist (CCLS) from the United States before being installed on wards. The intensive training (facilitated in English) consisted of input on CLS theory and supervised practical exercises. Furthermore, an ongoing training schedule has been implemented which covers theoretical input and practical sessions. CLSs were installed on wards with a focus on gastroenterological, metabolic, neurological and pulmonary diseases, as well as on the pediatric intensive care unit. Each CLS was assigned to one ward where s/he contacted and cared for patients and families. CLSs worked together with medical, nursing, and psychosocial employees (mainly educators and psychologists) and primarily undertook the following interventions: medical education and preparation, teaching of coping strategies, providing distraction and therapeutic play. The introduction of the CLS program was accompanied by information events outside the hospital (e.g., a children's health summit to raise awareness for the topic) as well as by regular meetings with physicians and nurses to inform about the new professional group. Overall, the implementation of the CLS program was prepared based on the experiences reported in model CLS programs from the United States and was constantly being adapted to the clinical hospital setting. The primary goal was to test and adapt the introduction of a pilot CLS program into the German health care system which could lead to a long-term and up-scaled introduction of such programs in Germany.

#### 2.2.2. Participants and recruitment

We recruited four groups of stakeholders for individual interviews: (i) patients (children 4–17 years), (ii) their parents, (iii) CLSs, and (iv) other health professionals with contact to the CLS program, e.g., physicians, nurses, educators. During recruitment, educators were identified as professionals with regular and close contact to CLSs: educators were present on all wards where CLSs worked and were discussing individual patient cases with CLSs in psychosocial team meetings. The psychosocial team in the hospital served as a gatekeeper for recruiting and provided the research team with a list of contact details of potential participants. Members of the psychosocial team were the only ones who had an overview of patients, parents and health professionals who already knew the CLS program. From this list, participants were recruited according to a purposeful and maximum variation sampling technique: patients from different age groups, with different diagnoses and on different wards; and health professionals with different professional backgrounds. CLSs were seen as multi-professionals who combined the experiences from their former professional background with their view in their new role as CLS. Due to contact and access restrictions during the COVID-19 pandemic, not all participants were contacted in person by the interviewer (JH), but via telephone or email. The researcher was already known to participants from the CLS team but had no contact to the other participants before the interviews. All participants were provided with extensive information concerning the purpose and procedure of the interview, the handling of the data, the interviewer's role in the project and her professional background. Age-appropriate information sheets and consent forms were used for children (three versions for patients aged 4–6, 7–13, and >14). All participants provided their written consent; for minors, the additional consent of their parents was required.

#### 2.2.3. Data collection

Semi-structured, guided interviews were conducted by one member of the research team (JH) between June 2021 and January 2022. For each group of participants (4–6, 7–13, >14-year-old patients; parents; CLSs; other health professionals) specific interview guides were developed based on the research aims, the CLS literature, the logic model and first practical experiences with the program (see Table 1 for excerpts of the interview guides). The interview guides were piloted paying particular attention to the age-appropriateness of the children's guides (i.e., children from the researchers’ circle of acquaintances with and without prior hospital experiences were asked to respond to a selection of interview questions and give feedback on intelligibility, scope and duration of the interview). The interview guides were designed to assess implementation processes through a set of different questions and topics: psychosocial care situation of children in the hospital; needs in the psychosocial care of children; experiences with CLSs in the daily routine of the hospital; expectations of CLSs. CLSs and hospital employees were additionally asked about the scope, potentials, and risks of CLSs’ work, and the implementation of the CLS program (development, distinction from and collaboration with other professional groups). Since this study focuses on factors influencing the implementation of the CLS program, only data and results concerning this topic will be reported (information on CLSs’ tasks, the scope of the CLS program etc. will be used internally for further development of the CLS program). Interview guides contained guiding questions, prompts and specific questions. Interviews were conducted in person in rooms of the hospital (patient's room, playroom, office), or via the secure videoconferencing software RED connect (26) depending on restrictions during the COVID-19 pandemic. Children could decide whether they wanted to conduct the interview in the presence of their parents or alone. All interviews were audiotaped and transcribed verbatim. Personal data was pseudonymized using numerical codes. Data collection was considered to be sufficient when themes in the material began to repeat, no new codes were generated and a profound understanding of thematic issues was developed (27), i.e., according to the principles of data saturation (28).

**Table 1:** Excerpt of interview guides for different groups of stakeholders.

**Children 4–6 years**
You/your parents have told me that you have already met … (name CLS). Can you tell me about your meeting with …? What did you do, what happened?
**Children 7–13 and 14–17 years**
You/your parents have told me that you have already met … (name CLS). Can you tell me about your meeting with …? What did you do, what happened?
Can you tell me what a typical meeting with …/a CLS looks like? What do you experience when you meet …/a CLS?
**Parents**
You and your child have already had contact with a CLS. Can you tell me about your experiences with him/her? From your experience, what does a typical contact with a CLS look like?
With regard to your experience with the processes and structures in the hospital, how did you experience the CLS within the hospital? How does the CLS fit into everyday hospital life?
**CLSs**
You are employed as a CLS and have been working in this “new” profession for some time now. Can you briefly describe in your own words what a CLS is?
How do you experience everyday life in the hospital as a CLS, specifically with regard to the novelty of your profession in this hospital, established processes and structures in the hospital, the cooperation with other professional groups?
**Health professionals**
You have already met or worked with a CLS here in the hospital. Can you briefly describe in your own words what a CLS is?
How do you experience the profession of CLS in everyday life in the hospital, specifically with regard to the novelty of the profession in this hospital, established processes and structures in the hospital, the cooperation with other professional groups?

CLS, Child Life Specialist. Only guiding questions concerning CLSs and the implementation of the CLS program are presented. The formulation of the questions was adapted to the individual interview partner.

#### 2.2.4. Data analysis

Data was analyzed using qualitative content analysis following Mayring's systematic approach to the interpretation of qualitative material based on defined analysis rules and steps (24). In this work, we focused on qualitative analysis methods without the use of quantitative steps such as the counting of frequencies of categories. Two authors (JH, VZ) read all interviews and developed a preliminary coding system. This system relied on elements deduced from the interview guide, the logic model, hypothesized concepts (i.e., factors influencing the implementation) as well as on information inductively gained from the material. Within the conceptual analysis of frameworks, we identified a good fit of our preliminary coding system with the three domains of barriers and facilitators for the implementation of patient-focused interventions in hospitals identified in a systematic review ((29); “system”, “staff”, and “intervention”). We therefore decided to categorize and present our results according to this literature-based framework. We treated all factors influencing the implementation as operating on a spectrum from acting as a barrier to acting as a facilitator since the distinction between barriers and facilitators proved to be inconclusive when working with the material (e.g., lacking communication was perceived as a barrier, whereas good communication was described as a facilitator). The preliminary version of the coding system was presented in an interdisciplinary methods’ workshop and refined based on discussions and feedback of participants. This revised coding system was then applied on two interviews which were coded separately by two authors (JH, VZ) to check the appropriateness and validity of the developed system. Discrepancies were discussed within the research team (MC, JH, CJ, VZ, SV) until consensus was reached and the coding system could be finalized. The remaining interview data was coded by one author (JH) with support of the research team when in doubt. Qualitative data analysis was performed in MAXQDA (30). All interviews were conducted in German. Similarly, all steps of the qualitative content analysis were performed in German. For this manuscript, all quotations were translated verbatim by one author (JH) and checked by a second author (native speaker, HC) in order to keep linguistic appropriateness while maintaining the original meaning. Throughout the whole process, researchers reflected potential biases and their consequences during data collection (e.g., potential sampling biases), and potential implications were discussed within the research team and considered during data analysis.

## 3. Results

### 3.1. Participants

Fifteen individual interviews were conducted with patients (*n* = 4), the parents of these patients (*n* = 4), CLSs (*n* = 4), and other health professionals (*n* = 3; educator *n* = 1; physicians *n* = 2). To maintain data protection and anonymity of one educator and two physicians interviewed, these professional groups were summarized under the term “health professional”. Table 2 shows the sample's characteristics and provides further information on the circumstances of the interviews. Most of the interviews (*n* = 9) took place face-to-face. The interviews with patients lasted between 15 and 20 min for younger children (5 and 8 years) and 45 min for the adolescent (17 years). The average duration of an interview with an adult was 44 min.

**Table 2:** Description of the sample and the circumstances of the interviews.

Participant	Duration	Format	Further information
Child 1	20	Virtual	Female, 8 years; hospitalized due to severe illness; was visited by a CLS several times a week during her stay; her father (Father 1) was present during the interview
Child 2	15	In presence	Male, 5 years; was hospitalized following an accident; was visited daily by a CLS during his stay; his mother (Mother 1) was present during the interview
Child 3	20	In presence	Male, 8 years; was hospitalized for an acute emergency; was visited daily by a CLS during his stay
Child 4	45	Virtual	Female, 17 years; was hospitalized due to a chronic illness; was visited daily by a CLS during her stay
Father 1	40	Virtual	Accompanied his daughter for the whole time during her hospital stay; met a CLS several times during the hospital stay
Mother 1	30	In presence	Accompanied her son for the whole time during his hospital stay; met a CLS daily during the hospital stay
Mother 2	40	In presence	Accompanied her son for the whole time during his hospital stay; met a CLS daily during the hospital stay
Father 2	45	Virtual	Visited his daughter during her hospital stays; regularly talked to and exchanged information with CLS
CLS 1	50	In presence	Working in the CLS program for 9 months at the time of the interview; professional background: teacher
CLS 2	45	In presence	Working in the CLS program for 3 months at the time of the interview; professional background: children's nurse
CLS 3	50	In presence	Working in the CLS program since the beginning (at the time of the interview for 14 months); professional background: children's nurse
CLS 4	50	In presence	Working in the CLS program since the beginning (at the time of the interview for 14 months); professional background: psychologist
Health professional 1	45	In presence	In contact with the CLS program and its team since the beginning (2020); working collaboratively with CLSs on individual patient cases
Health professional 2	45	Virtual	In contact with the CLS program and its team since the beginning (2020); working collaboratively with CLSs on individual patient cases
Health professional 3	40	Virtual	Working collaboratively with CLSs on individual patient cases since 2021

### 3.2. Factors influencing the implementation of the CLS program

We present our findings according to the three levels “system”, “staff”, and “intervention” (Table 3).

**Table 3:** Factors influencing the implementation of the CLS program on a system, staff and intervention level.

Level	Factors influencing the implementation
System	Definition of the CLS program
	Organizational culture
	Communication and integration
Staff	Training and perceived competence
	Individual skills
Intervention	Novelty
	Unique characteristic
	Flexibility
	Availability

#### 3.2.1. System

Factors influencing the implementation on a system-level relate to the structural and cultural context of the hospital, i.e., the setting where the CLS program is implemented (29).

##### 3.2.1.1. Definition of the CLS program

Participants, in particular CLSs and health professionals, repeatedly highlighted that the tasks of a CLS (as an unknown profession in Germany) should be clearly defined, and the scope of the new CLS program should be determined, preferably already before starting the implementation. In their opinion this had not happened to a sufficient extent.

*“It would have been helpful to consider what services were already being offered on the wards. We wanted to introduce something new but did not think about how our goals may coincide with others. The best strategy would have been to survey existing services and then work to complement them.” (Health professional 1)*

*“We needed more clarification regarding our offer of interventions in order to be more self-confident on the wards and to be able to say: ‘This is how we can help support the patient’.” (CLS 4)*

Additionally, participants reflected that the CLSs’ tasks should be more clearly delineated from those of other professional groups in the hospital. CLSs and health professionals pointed out that a lack of a clear distinction can result in an inefficient use of human resources by either not employing available CLS competencies or by certain activities being carried out by several professionals at the same time.

*“At the beginning it was difficult because no one knew who I was or what I was responsible for. I always waited for a call, but nothing ever came.” (CLS 3)*

*“It took some time before we were able to work together because our scope of activities seemingly overlapped. Initially, we were two people sitting there with two children trying to achieve similar goals and it was too much.” (Health professional 1)*

CLSs described the initial phase of the program as a challenging period. Feelings of being insufficiently prepared, not having enough knowledge as well as missing social networks led to stress. Getting to know the profession and the hospital's procedures were perceived as the main challenges when the CLS program started.

*“It was as if we had been abandoned on a desert island. We didn't know anyone on the wards because we did not have any contacts or previous relationships.” (CLS 4)*

##### 3.2.1.2. Organizational culture

The material in the interviews suggests that the attitudes of stakeholders about the profession of CLSs and the CLS program negatively impacted the implementation of the program. CLSs reported that they experienced refusal or negative remarks from other hospital employees during their visits on the wards. It was pointed out that the information on the CLS program which was disseminated before the actual program's launch created certain assumptions concerning the program and caused resistance in some hospital employees towards the CLS team and their work.

*“The others were thinking: ‘And what have we done so far? Where is the recognition for us?’ […] We want to work together and not against each other. That came across wrong. This immediately created resistance from the staff on the wards and made it difficult for others to accept us.” (CLS 3)*

*“One problem is that [the CLS program] is not accepted by everyone. […] I have noticed that some doctors and nurses do not see the benefit [of the CLS program] or do not value it as much.” (Health professional 3)*

Furthermore, CLSs were excluded from internal teams due to missing knowledge about or acceptance of the program.

*“There was limited communication and understanding about what our role was on the wards or how to reach us. Many asked questions like: ‘What are you doing here again?’, and when we informed them, they told us: ‘You will just have to wait and see how to get involved because we don't have time to call you’.” (CLS 4)*

CLSs tried to explain negative attitudes with a fear of losing competencies: other professional groups may have concerns about having to hand over their own activities and areas of responsibility to CLSs which is why they may have been reluctant to integrate CLSs in internal networks or share knowledge and experiences.

*“We were attempting to integrate a new service into a long-standing system, which I think is always difficult, regardless of the hospital. Our scope of practice needed to be better defined in order for others to understand how we could help and to reduce concerns about losing own responsibilities.” (CLS 1)*

According to CLSs, refusing attitudes concerning the CLS program could be reduced by the perception of the program as relevant and meaningful. CLSs stated that the other health professionals first needed to make their own experiences with CLSs’ work. When there was an understanding of the goals of the program as well as a perception of possible benefits, acceptance of the CLS program could be achieved.

*“Thank goodness [the resistance] lifted after they realized what we do and that it can positively make a difference. This helped us work together without conflict and it works quite well now.” (CLS 3)*

##### 3.2.1.3. Communication and integration

According to participants, the collaboration with other hospital employees affected the integration of the CLS program into the hospital. Almost all interviewed professionals noted that good communication (within the CLS team as well as with other hospital employees) is an important requirement for the integration of the CLS program into the hospital. The exchange of information can reduce prejudices and resistance. Furthermore, participants thought that communicating responsibilities could strengthen mutual support between CLSs and other hospital employees so that tasks would be divided, and the workload would be reduced.

*“If you communicate well, then it doesn't matter which professional group you work with. It’s just a question of communication. (…) It’s always like that when something new comes along, but I think you can solve that by establishing a good contact and by communicating areas of responsibility.” (CLS 2)*

*“Clear communication can make a big difference. When they realize we have a clear goal, are following a plan and document our interventions in the patient’s chart for them to read, then they know: ‘Aha, the CLS is providing a specific service, which is not my job at all and it’s good this gap is being filled.’ I think with time they will understand.” (CLS 3)*

Although communication and integration processes had already started, CLSs expressed their wish for expanding this development and being more directly involved with other professional groups.

*“Success is when you are asked in advance to support a child on the ward. For example: ‘Could you provide medical education for…?’, or: ‘We are having this problem with this child, can you assist?’. This would prove that we are more accepted and more involved.” (CLS 2)*

#### 3.2.2. Staff

Factors influencing the implementation on a staff-level relate to the personal characteristics and skills of those who carry out the CLS program, that is the CLSs themselves (29).

##### 3.2.2.1. Training and perceived competence

Although various positive experiences in the interaction with CLSs were reported, the CLSs themselves felt they had not been trained appropriately. CLSs didn't feel sufficiently skilled and described that they mainly had to rely on their previous personal and professional competencies. The interviewed CLSs expressed their wish for (more) training sessions and further CLS-specific qualification.

*“We didn’t have any guidelines to follow. We had to build everything ourselves: develop concepts and prepare ourselves.” (CLS 3)*

*“Because CLS is a new professional field in Germany, there was no available fixed training, so sometimes we lacked education for simple situations.” (CLS 1)*

##### 3.2.2.2. Individual skills

In general, patients and parents valued the CLSs’ interaction with patients and felt CLSs were able to build reliable relationships with them and their families. According to patients and parents, compared to other health professionals CLSs obtained more information and were provided with deeper insights into patients’ and parents’ needs and worries.

*“[The CLS] is very close to the children, much closer than a doctor, and most of the time even closer than the nurses. This is because [the CLS] spends so much time with the children, plays with them, and talks with them in a language they understand. She notices what they are afraid of.” (Child 4)*

*“[The CLS] came and started playing [the patient’s] favorite game half an hour before the blood draw, so that he was already into the game during the blood draw. That was great. Because the [other professionals have] no time for that.” (Mother 2)*

#### 3.2.3. Intervention

Factors influencing the implementation on an intervention-level relate to the characteristics and components of the intervention that support or hinder the implementation of the CLS program (29).

##### 3.2.3.1. Novelty

The characteristic of the CLS program as being new and unknown within the hospital context was commented on by many interviewees. Patients and parents reported that they were neither familiar with the term “Child Life Specialist” nor with the associated tasks of a CLS. Furthermore, the English term “Child Life Specialist” was difficult to pronounce and remember for a German speaker, especially for children. One patient, although having a close relationship to a CLS, did not use the term “Child Life Specialist”.

*Interviewer: “Do you know what kind of job [the CLS] has here?”*

*Child: “She is a child guardian [literally “Kinderbetreuerin”], right?” (Child 3)*

Parents pointed out that for them it was difficult to understand the CLSs’ role, particularly at the beginning of the hospital stay when they needed to gain a general understanding of the hospital's routines and when their stress-level was especially high. It took time to locate the CLSs and their tasks within the hospital context.

*“At the beginning, I couldn’t tell what [the CLS] was responsible for. Everyone introduces themselves, but you don’t really know who they are and what they do at first.” (Mother 1)*

The lack of knowledge on the work of CLSs did not only apply to patients and parents, but also to the employees in the hospital, as one CLS stated.

*“I once sent the doctors a list of my areas of responsibility and when I was available because they didn’t seem to know what my role was. Now they know when they can reach me, and that I will come right away, but many things are still unclear.” (CLS 3)*

##### 3.2.3.2. Unique characteristic

When asked about the CLSs’ tasks, patients, and parents as well as other health professionals answered that CLSs were responsible for preparing patients for medical procedures. According to participants, the ability to prepare patients for medical procedures using methods that are appropriate to the patient's age and developmental stage is a unique characteristic of CLSs which delineates them from other employees in the hospital. Generally, parents felt CLSs had specific competencies and medical knowledge that other psychosocial professionals did not have.

*“We should pay more attention to the fact that it is not only the parents who need to be educated and prepared for medical procedures, but also the children. […]. In my opinion, that is the goal of the CLSs’ work.” (Health professional 3)*

*“The CLS can fill the gap, if for example, we don’t understand something or if we don’t dare to ask the doctor, [the CLS] can often help. […] In my experience, she has a deeper understanding about medical procedures than the educators or psychologists.” (Father 1)*

##### 3.2.3.3. Flexibility

The CLS program being flexible and individualized increased the acceptance for the program. Patients and parents strongly valued that the CLSs were to a large extent able to consider patients’ needs and adapt their work to them. CLSs seemed to constantly evaluate the patients’ individual situations and to adopt the frequency of their contacts and the kind of intervention depending on what was needed most. Parents perceived this flexibility in the CLSs’ work, which contrasts the work of other health professionals, as a significant advantage. They felt CLSs were more detached from hospital structures and tight schedules and therefore more independent to fill emerging gaps in health care. Additionally, according to patients and parents, CLSs seemed to dispose of more time and appeared less stressed than other health care professionals which further supported the CLSs’ flexible and independent mode of working.

*“[The CLS] has always tried to visit me as often as possible. Even if it was only for a short time. Then she came in and said: ‘I don't have much time now, but I'll come again this afternoon.’ […] And she has the possibility to adapt her program or what she does to the age [of patients].” (Child 4)*

*“Perceived from the outside, [the CLSs] have a bit more freedom, what they take care of, where and when. This may also give them the opportunity to fill gaps that may otherwise occur in the patients’ care […]. I think it’s important to have someone not so strictly tied into the structures as the others who work [in the hospital]. Someone who can act a bit more flexibly.” (Father 2)*

Similarly, a CLS, formerly working as a children’s nurse, defined the higher amount of time available for CLSs as an essential and differentiating aspect of CLSs’ work.

*“In general, I think that [the CLS tasks] would be what I have been doing all along as a children’s nurse, except that I simply have more time for it, because that’s what’s missing in nursing. That you can simply take your time with the patients to work out solutions to problems, have conversations, or have the time to distract them during difficult examinations.” (CLS 2)*

##### 3.2.3.4. Availability

CLSs and other health professionals reported that the CLS program and the profession of CLSs *per se* were not sufficiently present within the hospital. According to the interviewees, a constant and reliable availability could hardly be established due to a small number of employees, frequent changes in staffing and long periods of time when a CLS was either not available at all on a ward or only available for a very short period of time and thus unable to address all demands. CLSs and other health professionals felt that this could have caused resentment, weakened professional networks, and consequently reduced the program's acceptance.

*“If there are lean periods, this is bad for the [CLS] project. If a structure to which one has become accustomed changes constantly or if a CLS is sometimes present and sometimes not at all, then one cannot rely on them.” (Health professional 2)*

*“We need more staff to succeed. The current situation doesn’t allow us to do anything well and because we are always in short supply, it creates difficult situations because we were somehow not there for half a day or a day again. People are extremely demotivated because of this.” (CLS 4)*

## 4. Discussion

### 4.1. Key findings and locating them in the literature

This process evaluation of a newly introduced pilot CLS program in a German children's hospital revealed key factors influencing the implementation on three levels (system, staff and intervention). On the system level, a clearer definition of CLSs’ tasks was perceived as important and would likely lead to a delineation from other professions and to a reduction of potential resistances towards the new program. On the staff level, lacking training opportunities and feelings of being insufficiently skilled were limiting the CLSs professional self-confidence. Finally, on the intervention level, the emergence of a unique characteristic of the CLSs’ work (i.e., preparation for medical procedures) supported the acceptance of the new program.

#### 4.1.1. Factors influencing the implementation on a system level

Since there is not much research on the implementation of CLS or other pediatric psychosocial care programs in children's hospitals, we compared our results with studies examining factors influencing the implementation of new interventions in a broader hospital setting. As in our findings, other qualitative studies report negative attitudes of hospital employees towards new interventions either based on resistance due to misinterpretations, on fatigue towards new initiatives, or on role conflicts and competing priorities (31, 32). Negative feelings towards and a lack of understanding for the CLSs’ tasks is also reported in a qualitative study on nurses’ perceptions on the CLSs’ influence on their work (33). Similarly, effective communication processes are commonly regarded as an important facilitator for implementation since relevant knowledge on new interventions can be disseminated and unrealistic expectations can be reduced (34). As soon as positive outcomes and long-term benefits of an intervention are perceived, negative attitudes and conflicts can be reduced (35), similar to the judgements by our interviewees. In terms of implementation outcomes (36), *acceptability* of a new program is important to assess and can strongly influence implementation.

Support and commitment from the hospital's management can facilitate the implementation of new interventions, which is also shown in a systematic review on the diffusion of innovations in healthcare (37). Additionally, changing established hospital structures and procedures can facilitate the implementation of a new intervention and should be promoted by leaders and local champions, according to a qualitative study on factors influencing the implementation of a new clinical pathway in Australia (38). Our results also indicate that managerial support is relevant for implementing the new CLS program within an existing system, most notably concerning the determination of the scope of the program and the delineation from other professions in the hospital. Setting the organizational framework in which a new professional group can operate should be part of the support and commitment of the hospital's management and could signalize an important implementation strategy for such new programs.

#### 4.1.2. Factors influencing the implementation on a staff level

A lack of confidence and skills of employees can impede the implementation process of new interventions which is also shown in a qualitative study on the implementation of a new outcome measure in palliative care in the United Kingdom (39). This finding is in line with our study in which CLSs reported feelings of not being sufficiently trained and lacking confidence when working on the wards. This lead to a high (mental) burden experienced by CLSs. Although the CLSs have been trained by a Certified CLS at the beginning of the program, training opportunities were limited due to language barriers (English speaking Certified CLS from the United States in a German hospital), contact restrictions during the COVID-19 pandemic and time constraints (CLSs needed to fulfill many tasks at the same time). Nevertheless, if employees feel they are skilled with regards to everyday requirements their stress levels can be reduced, and change can be facilitated (39).

#### 4.1.3. Factors influencing the implementation on an intervention level

Participants in our study reported that CLSs had a positive impact on patients, parents and health care professionals, but CLSs themselves did not perceive this as such. According to patients and parents, CLSs were particularly competent in building trustful relationships and collecting relevant information on individual patients, often going beyond the information available for other professional groups. Furthermore, the CLSs’ ability to provide medical education and preparation was particularly appreciated by patients and parents. A positive impact of CLSs on different stakeholders is reported in a qualitative study on nurses’ perceptions on the CLSs’ influence on their work (33). Nurses valued the additional time of CLSs, their contribution to the medical preparation of patients and felt relieved by the CLSs’ efforts. The CLSs in our study with a professional background as children's nurses similarly described the higher amount of time available for CLSs as an essential feature delineating both professions. However, the CLSs in our study were not aware of their special qualifications and their added value or did not perceive them as such. A focus on and the recognition of existing qualities could strengthen the CLSs’ position within the hospital system. Additionally, qualitative evidence on the implementation of new hospital processes states that providing further training opportunities could also act as a facilitator for implementation (34). In general, responding to the employees’ needs and involving them in decisions can raise their engagement with the new program, increase their empowerment and finally facilitate change within an existing system (38).

Furthermore, our participants reported that the novelty of the CLS program in the German health care setting and the missing knowledge on tasks of CLSs led to difficulties in understanding the CLS program in patients, parents, and hospital employees. In terms of implementation outcomes (36), the *appropriateness* of the CLS program (i.e., the fit of this new program in a new setting) was limited particularly at the beginning of the program which was a barrier for the implementation. This is in line with other qualitative studies highlighting that a successful implementation of a new program requires a good fit between the program and existing hospital structures (40). A good fit will only be achieved if the intervention is flexible enough to adapt to an existing system and/or the system is open for integrating new agents and interventions. Establishing a good fit becomes more difficult if a program is complex. In such cases, a successful implementation requires adaptations on various levels which increases the likelihood for potential conflicts and implementation problems. Although participants in our study complained about the low level of CLSs’ integration within hospital structures, this may have allowed CLSs to operate more independently and to flexibly adapt their interventions to the patients’ individual needs. The acceptance of a program by patients as well as the perception of a new program as producing positive patient outcomes were identified as important facilitators for the implementation of patient-focused interventions in hospitals (29). One component of the CLS program for which participants reported high acceptance and as being a good fit within the hospital context was medical education and preparation by CLSs. This component was regarded as a unique characteristic of CLSs, which helped delineate CLSs from other psychosocial professionals and supported the CLSs’ acceptance and their perceived suitability within the hospital.

### 4.2. Recommendations and future perspectives

From the material of our interviews practical implementation strategies can be deduced regarding the introduction of a new CLS program within an established hospital setting. The data from the interviews highlights the relevance of guiding the acceptability and integration of a new program. Specifically, our data indicates that for the launch of the new program it is important to prepare the environmental context within the hospital (i.e., structures, processes, employees), as well as to adapt the program to the specific organizational context in a hospital. The variety of role titles, responsibilities and specific interventions undertaken by CLSs (and comparable professions) identified in a scoping review (7) may indicate that different contexts require different elaborations of CLS programs. Furthermore, the definition of tasks of CLSs as a new professional group in the health care setting is relevant (e.g., in form of a manual). This also includes the delineation from other professions and the description of unique characteristics. This information should be disseminated through a well-conceived information campaign employing multiple communication channels and approaches within the hospital.

Our findings indicate that the implementation of a new complex CLS program is a challenging process. Therefore, implementation strategies of reducing complexity of the intervention itself and the CLS program as a whole may be facilitators. For instance, each CLS could be firmly assigned to a specific unit and integrated into all processes. This could provide clarity about the CLSs’ scope of action and facilitate better integration within the team due to constant availability and increased contact. A further reduction of complexity could be achieved if CLSs were not only assigned to a specific job site but if they also focused on distinct interventions. These should be selected based on an analysis of needs and demands, with the aim to strengthen children's perspectives as well as in distinction to existing services. A reduction of CLSs’ scope of action to a certain site and to specific tasks could sharpen the CLSs’ job profile and raise the confidence of CLSs in their own work. The range of tasks and responsibilities can be expanded in a next step when awareness and acceptance for the new program are established, and first implementation processes proved to be successful.

The difficulties in introducing a CLS program into the German pediatric health care system may be due to the fact that other pediatric psychosocial care services already exist. In the current care system considering children's needs in all clinical processes is not sufficiently taken into account (10), and other CLS-related aspects may also be underrepresented (e.g., preparing children for medical procedures). However, the introduction of the new profession of CLSs could lead to a duplication of tasks and conflicts of competence. Introducing a new profession is only one way to overcome current challenges in pediatric health care. It needs to be considered whether identified gaps could be closed more effectively by introducing a new profession (while facing associated challenges) or by sharpening existing professions and assigning additional tasks to them.

### 4.3. Strengths and limitations

Our study had several strengths. The interview guide was (i) developed according to rigorous methodological approaches and finalized based on consensual decisions within the research team, (ii) discussed in an interdisciplinary method's workshop, and (iii) piloted with regards to its appropriateness, particularly for use with children. A range of different stakeholders were interviewed, also including the perspective of children and adolescents in their role as patients, as well as CLSs as multi-professionals who combined the experiences from their former professional background with their view in their new role as CLSs. Data analysis was conducted in accordance with established methods of qualitative content analysis (24). The coding system was developed by two independent researchers and its validity was discussed within the research team.

Our study also has limitations. The recruitment was based on contacts of the psychosocial team in the hospital which may have led to sampling biases. However, this couldn't be avoided since members of the psychosocial team were the only ones who had an overview of potential participants who already had contact to the CLS program. The research team who conducted the process evaluation was in regular contact and exchange with the hospital team responsible for the implementation of the CLS pilot. Therefore, the research team was informed about the ongoing implementation process and associated challenges while designing the process evaluation. Additionally, the interviewer (JH) already knew the interviewed CLSs before the study. While facilitating recruitment and an open communication, prior personal relationships may also have influenced the interviewees’ responses. On the other hand, there was no personal contact prior to the study to the other interviewees (patients, parents, health professionals). The researchers tried to mitigate the effects of potential biases through regular reflections and discussions in the interdisciplinary team with extensive experiences in qualitative methods. Moreover, interviews were conducted during contact restrictions due to the COVID-19 pandemic which (in some cases) limited possibilities for relationship building. Particularly, the interviews with younger children would have benefited from personal interactions (in presence) before starting the conversation. The COVID-19 pandemic additionally complicated the recruitment of hospital employees. In the highly structured hospital routine with an extreme workload, which was exacerbated by the COVID-19 pandemic, three health professionals (one educator, two physicians) were available for interviews, and no participant could be recruited from the nursing professions, despite several attempts to make contact. However, two of the interviewed CLSs had a professional background as children's nurses which we consider representatives of this perspectives in our material. Nevertheless, we reached data saturation during coding and data analyses.

## 5. Conclusions

The implementation of a new professional service focusing on pedagogical aspects and the respect for children into an established hospital system is challenging and supporting structural guidelines are lacking. Our data highlights the importance of preparing the context as well as developing a clear job description. This should be based on the needs of the target groups and identified gaps in the health care system. Our results contribute to a better understanding of processes during the implementation of a new professional service and provide practical recommendations for meeting emerging challenges. The experiences of this pilot CLS program could support a structured implementation of (long-term and larger) CLS programs in Germany and other contexts. More research in other settings is needed to consolidate our results and extend scientific knowledge on implementing complex interventions into hospital settings.

## Data Availability

The datasets presented in this article are not readily available because patient confidentiality and participant privacy must be maintained. Excerpts from the interview guide are presented in the manuscript. Requests to access the datasets should be directed to Julia Hummel, jhummel@ibe.med.uni-muenchen.de.
